# A System for Real-Time, Online Mixed-Reality Visualization of Cardiac Magnetic Resonance Images

**DOI:** 10.3390/jimaging7120274

**Published:** 2021-12-14

**Authors:** Dominique Franson, Andrew Dupuis, Vikas Gulani, Mark Griswold, Nicole Seiberlich

**Affiliations:** 1 Department of Biomedical Engineering, Case Western Reserve University, Cleveland, OH 44106, USA; mark.griswold@case.edu; 2 Department of Radiology, University of Michigan, Ann Arbor, MI 48109, USA; vikasgulani@med.umich.edu (V.G.); nse@med.umich.edu (N.S.); 3 Department of Radiology, Case Western Reserve University, Cleveland, OH 44106, USA

**Keywords:** real-time imaging, mixed reality, magnetic resonance imaging, GRAPPA, non-cartesian, interventional guidance

## Abstract

Image-guided cardiovascular interventions are rapidly evolving procedures that necessitate imaging systems capable of rapid data acquisition and low-latency image reconstruction and visualization. Compared to alternative modalities, Magnetic Resonance Imaging (MRI) is attractive for guidance in complex interventional settings thanks to excellent soft tissue contrast and large fields-of-view without exposure to ionizing radiation. However, most clinically deployed MRI sequences and visualization pipelines exhibit poor latency characteristics, and spatial integration of complex anatomy and device orientation can be challenging on conventional 2D displays. This work demonstrates a proof-of-concept system linking real-time cardiac MR image acquisition, online low-latency reconstruction, and a stereoscopic display to support further development in real-time MR-guided intervention. Data are acquired using an undersampled, radial trajectory and reconstructed via parallelized through-time radial generalized autocalibrating partially parallel acquisition (GRAPPA) implemented on graphics processing units. Images are rendered for display in a stereoscopic mixed-reality head-mounted display. The system is successfully tested by imaging standard cardiac views in healthy volunteers. Datasets comprised of one slice (46 ms), two slices (92 ms), and three slices (138 ms) are collected, with the acquisition time of each listed in parentheses. Images are displayed with latencies of 42 ms/frame or less for all three conditions. Volumetric data are acquired at one volume per heartbeat with acquisition times of 467 ms and 588 ms when 8 and 12 partitions are acquired, respectively. Volumes are displayed with a latency of 286 ms or less. The faster-than-acquisition latencies for both planar and volumetric display enable real-time 3D visualization of the heart.

## 1. Introduction

Image-guided cardiovascular interventions, such as catheterizations and electrophysiology procedures, require an operator to precisely maneuver devices and visualize structures of interest in areas that require continuous, real-time monitoring due to blood flow, cardiac motion, respiratory motion, or other physiological motion. Ultrasound and fluoroscopic X-rays are commonly used to guide these procedures and can routinely image at 30 frames per second (fps) with minimal display latency [1,2]. However, both modalities face limitations in their interventional utility: ultrasound provides only small fields of view and limited depth penetration, and fluoroscopy yields poor soft tissue contrast and non-visualization of tissue surrounding opacified structures and requires the use of ionizing radiation. Specialized device localization systems have also been developed, such as electrophysiology mapping systems that are regularly used for cardiac ablation [3]. However, these systems are application-specific and may require purpose-built devices. Magnetic Resonance Imaging (MRI) is increasingly being explored as a promising modality for cardiovascular interventions because it provides excellent soft tissue contrast, does not use ionizing radiation, and can obtain planar and volumetric images in oblique orientations with large fields of view [4,5,6,7]. However, most clinically deployed MRI protocols for cardiovascular interventional guidance are relatively slow compared to ultrasound and fluoroscopy.

### 1.1. Real-Time Magnetic Resonance Imaging and Reconstruction Protocols

Interventional guidance for cardiovascular procedures requires rapid data acquisition paired with low-latency image reconstruction with inline image display (i.e., images that are available at the MRI scanner). Clinically deployed MR guidance for cardiovascular interventions is typically performed with one to three planar slices, acquired at a frame rate of 5.5 to 13 fps and a resolution of 2 to 3 mm^2^ in-plane with a 8 to 10 mm slice thickness [8,9,10,11,12,13]. Imaging is often performed with Cartesian trajectories and may use data undersampling with acceleration factors of 2 to 4. If the data are undersampled, parallel imaging methods, such as sensitivity encoding (SENSE) [14] or generalized autocalibrating partially parallel acquisition (GRAPPA) [15], can be used to recover the images. However, more rapid data acquisition techniques that are already available in MRI are not often utilized clinically because they require advanced reconstructions that can be difficult to implement with low latency. Additionally, when imaging with only a few planar slices, an operator may need to regularly reposition the slices to continuously track devices and target tissues, a process that requires a skilled understanding of how a device may move within the anatomy. Volumetric imaging may provide visualization of all organs and devices within the region of interest and obviate the need for regular slice repositioning, but the data acquisition and reconstruction times are significantly longer than for planar imaging. Thus, rapid data acquisition coupled with a low-latency implementation of an appropriate reconstruction method may enable planar imaging with a spatial resolution comparable to current protocols but with a higher temporal resolution, and it may also enable the exploration of rapid volumetric imaging guidance.

Non-Cartesian trajectories and high data undersampling rates have previously been deployed to significantly speed up MRI data acquisition, and a number of different image reconstruction techniques have been proposed to generate images from highly accelerated non-Cartesian data [16,17,18,19,20,21]. Through-time radial GRAPPA is one such technique that can enable acquisitions of 14 to 28 fps (35 to 70 ms/frame) for a single cardiac slice with spatial resolutions of 1.56 to 1.73 mm^2^ [22]. This reconstruction has also enabled volumetric cardiac imaging with a temporal footprint of 341 ms and a spatial resolution of 2.3 × 2.3 × 8 mm^3^ [23].

While other groups have proposed non-Cartesian acquisitions coupled with rapid, inline reconstruction frameworks for procedure monitoring [24,25,26,27], these methods do not use a non-Cartesian GRAPPA technique for real-time reconstruction. Several features of through-time radial GRAPPA make the reconstruction amenable to low-latency implementations. Because temporal information is not incorporated (information is not shared across accelerated frames), each frame can be reconstructed immediately following data collection for the frame. The calibration process, in which the GRAPPA weights are calculated, can be performed as a separate step prior to reconstructing the undersampled frames. A set of weights can then be used to reconstruct many repetitions of accelerated data, as long as the imaging slice’s position and orientation do not change. Importantly, the technique is not iterative, and the process of estimating unacquired data points can be can be parallelized using graphics processing units (GPUs) such that the reconstruction is as fast as or faster than the data acquisition. A real-time, inline implementation for planar imaging with radial GRAPPA has previously been developed by Saybasili et al. using a heterogeneous architecture with multi-core central processing units (CPUs) and GPUs [28]. However, it would be beneficial to have an implementation for both planar and volumetric imaging on an open-source platform that can utilize existing, specialized image reconstruction libraries. Recently, several frameworks have been developed to promote vendor-agnostic and open-source MR reconstruction code [29,30,31]. The Gadgetron framework is one example that provides several reconstruction modules that are useful for real-time, non-Cartesian imaging, and it can be deployed at the scanner for inline reconstruction [29].

### 1.2. Medical Imaging Visualization Approaches

One advantage of MRI is that data can be acquired as multiple oblique, planar slices as well as contiguous volumes. However, current display techniques utilizing 2D monitors may not present oblique or volumetric data in a manner that facilitates rapid interpretation of device position and anatomy for interventions. For example, multiple oblique slices are often shown side by side, which obscures their spatial relationship with respect to one another. This can be partially resolved by transforming multiple slices to intersect each other in a 3D coordinate system. However, this necessitates monoscopic projection for use with a 2D display and collapses the spatial information present. Virtual-reality (VR) and mixed-reality systems have been proposed by several groups as alternatives to standard 2D displays of MR images because they offer the ability to render stereoscopic projections of the data, retaining additional spatial context [32,33,34,35,36,37,38,39,40].

Although VR may provide an immersive experience for applications, such as patient education, a mixed-reality system is more suitable for medical interventions. In mixed reality, the user retains the ability to see and interact with the physical surroundings. Specifically, the interventionalist would see both the translucent stereoscopic projections and the natural environment. The patient and access areas remain visible, and the interventionalist can keep track of devices and the operating table, monitor equipment, and work seamlessly with others in the room. While systems utilizing MRI-based mixed reality for procedural guidance have been proposed by a few groups [41,42,43], to the best of our knowledge, there are not yet any systems that combine MR imaging rapid enough to guide cardiac interventions with faster-than-acquisition, stereoscopic display in mixed reality.

### 1.3. Research Aims

**Figure 1:**
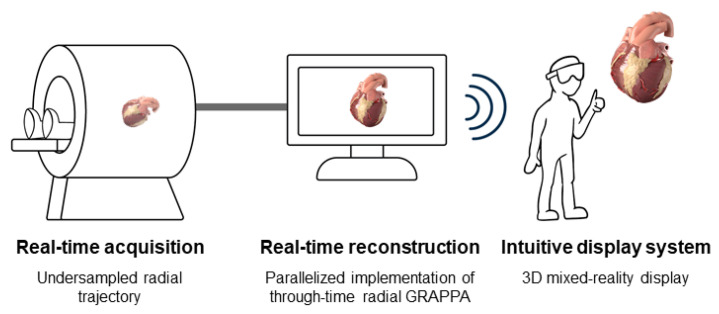
Concept illustration of the real-time, online mixed-reality visualization system. MR data are collected using an undersampled, radial trajectory for rapid acquisition. Real-time image reconstruction is performed using a parallelized implementation of through-time radial GRAPPA. The final images are rendered in a mixed-reality headset for intuitive display.

The goal of this work was to develop a system for cardiac MR imaging that operates in real-time at the scanner and presents images in an intuitive format, with the future goal of providing guidance for cardiovascular interventional procedures [44]. Data are acquired using an undersampled radial trajectory and reconstructed using a GPU-parallelized implementation of through-time radial GRAPPA in the Gadgetron framework. A user wearing a Microsoft HoloLens headset sees the MR images as 3D mixed-reality renderings that are updated in real-time to reflect the current status of the person being scanned. Figure 1 shows a concept illustration of the proposed system. “Real-time” here is defined as planar data acquired with a spatial resolution comparable to current cardiac MR imaging for interventional procedures but with faster temporal resolution (>13 fps or 77 ms/frame) and approaching that offered by fluoroscopy (30 fps or 33 ms/frame) and volumetric data acquired within one heartbeat. Images are presented with a display latency that is shorter than the data acquisition time. We describe the set-up and testing of the data collection, image reconstruction, data transfer, and rendering for this real-time MRI mixed-reality system for cardiac imaging.

## 2. Materials and Methods

**Figure 2:**
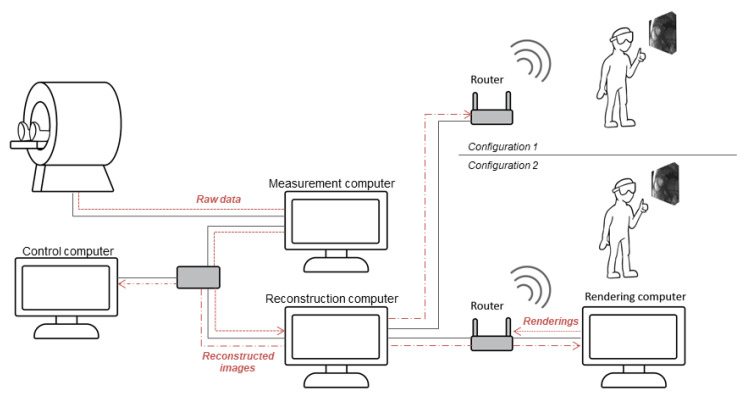
Schematic of data flow within the online acquisition, reconstruction, and mixed-reality rendering system. A dedicated image reconstruction computer was introduced into the network containing the scanner, the scanner control computer, and the scanner measurement computer via a switch and an Ethernet cable. A second network was set up using a router to contain the reconstruction computer and the mixed-reality headset. Two configurations were possible. In the first, reconstructed data are sent directly to a self-contained HMD capable of handling the entire rendering pipeline. In the second, a dedicated rendering workstation is used. The reconstruction and rendering computers are connected via Ethernet cables, and rendered frame data are transferred wirelessly to the headset. Solid gray lines show component connections, and dashed red lines depict data transfer.

The system was designed to operate at the scanner control console. Figure 2 shows the major hardware components, data pathways, and local networks of the system. Undersampled, raw data are transferred from the scanner measurement computer to the reconstruction computer, where the through-time radial GRAPPA reconstruction is applied in the Gadgetron framework [29] and the multi-channel data are converted to images. These images, as well as their position and orientation vectors within the scanner reference frame, are then transferred to the scanner control computer for display in the standard display window, as well as to a rendering machine of some kind. The rendering machine is responsible for spatial orientation and stereoscopic display of the image data, as well as managing window, level, and other GUI considerations. Rendering machines currently supported by the pipeline can be either a self-sufficient mobile HMD, such as the Microsoft Hololens 2, or, alternatively, a more powerful discrete workstation from which the rendered data are transmitted to a less capable wireless headset for presentation in mixed reality.

### 2.1. Data Acquisition

All data were acquired at 3T (Skyra, Siemens Healthineers, Erlangen, Germany) on an imaging phantom and on healthy volunteers according to an IRB-approved protocol. Depending on the number of image slices and the slice positions, 30 to 34 coils were used to receive the signal. An undersampled radial trajectory with an acceleration factor of 9 (16 out of 144 radial projections acquired) was used in a spoiled gradient echo sequence. With the scan parameters listed below, this acceleration factor enabled acquisition times of 46 ms per planar slice, 467 ms per 8-partition volume, and 588 ms per 12-partition volume.

The following scan parameters were used for planar imaging: field-of-view = 300 × 300 mm^2^; matrix size = 128 × 128; in-plane resolution = 2.34 × 2.34 mm^2^; slice thickness = 8 mm; flip angle = 7° to 12°; repetition time (TR)/echo time (TE) = 2.88 ms/1.51 ms; bandwidth = 1000 Hz/pixel; readout oversampling factor = 2. One to three slices were acquired per frame, and slices could be oriented arbitrarily relative to each other to cover common cardiac MRI views, such as short axis, three chamber, four chamber, left ventricular outflow tract, and aortic arch views. Data were acquired continuously in an ungated, free-breathing fashion.

Volumetric imaging was performed with the same parameters as for planar imaging with the following modifications: partition thickness = 8 mm (8 partitions) or 5 mm (12 partitions); TR/TE = 2.92 ms/0.73 ms (8 partitions) or 3.06 ms/0.73 ms (12 partitions); bandwidth = 1115 Hz/pixel. Datasets were centered over the left ventricle in a short-axis orientation with 8 or 12 partitions. The data were acquired in a stack-of-spokes configuration, and undersampling was performed only in-plane. A partition oversampling factor of 25% (8 partitions) or 16.7% (12 partitions) was used, and a partial Fourier factor of 7/8 was applied if 12 partitions were acquired. The data were collected during free-breathing using electrocardiogram (ECG) gating to acquire in diastole.

Prior to acquiring the undersampled data, 60 (planar) or 10 (volumetric) fully sampled frames of data were collected in an ungated, free-breathing acquisition as calibration data for the through-time radial GRAPPA reconstruction. This calibration data collection phase was 25, 50, or 75 s for planar imaging of one, two, or three slices, respectively, and 42 or 62 s for volumetric imaging of eight or twelve partitions.

In addition to in vivo scanning, data were acquired in imaging phantoms filled with a nickel-sulfate solution (spherical and bottle phantoms, models 10496625 and 08624186, Siemens Healthcare, Erlangen, Germany) for timing measurements. Five hundred frames of one-, two-, and three-slice planar data and eight- and twelve-partition volumetric data were acquired using the same parameters as for in vivo scanning, while imaging in a phantom allowed for long scans to measure the stability of the system over time. Volumetric data were acquired with a simulated heart rate of 75 bpm.

### 2.2. Image Reconstruction

Through-time radial GRAPPA ported into the Gadgetron framework [29] was used for fast, inline reconstruction [45]. Two separate pipelines for the calibration and reconstruction processes were created. The computation-intensive calibration process was performed on the CPU, and the resulting GRAPPA weights were saved. In the reconstruction pipeline, the GRAPPA weights were loaded into the pipeline and transferred to the GPU, and the parallelizable target point estimation (where each point is estimated independently) was performed on the GPU. Note that the weights are read in and moved to the GPU only on the first call to the pipeline (when the first repetition is processed). The GRAPPA reconstruction was performed with a GRAPPA kernel size of 3 × 2 (readout × projection) and a segment size of 8 × 1 (readout × projection), as in previous work [22]. A GPU-accelerated nonuniform fast Fourier transform (NUFFT) algorithm available in the Gadgetron toolbox [46] was used to resample the reconstructed radial data onto a Cartesian grid and generate images. Density compensation weights were calculated using Voronoi polygons using a utility function also available in the Gadgetron framework. Single-coil images were then combined using sum-of-squares to yield a single image. The final images were cropped to a 90 × 90 matrix size around the heart for ease of viewing and interpretation.

At the beginning of both the calibration and reconstruction pipelines, the readout oversampling was removed, and a principal component analysis (PCA)-based coil compression step to 12 virtual coils was performed. These processing steps were used to reduce the time required for the downstream steps, primarily the GRAPPA reconstruction and the NUFFT.

All testing was performed in Gadgetron version 3.14.1.

### 2.3. Rendering

Once real-time images have been reconstructed, the next step is to arrange them in context and render them for mixed-reality display. A naïve ray marching algorithm was used for volume rendering, and a standard two-sided texture shader was used for planar slices [47].

Planar slices were rendered at their relative spatial locations using the position and orientation vectors recorded for each dataset by the MRI scanner. Although multi-slice planar data were acquired and reconstructed in a slice-by-slice fashion, all of the slices in the rendered frame were updated at once to provide a smoother visualization. Note that the user is able to switch to updating the frame slice-by-slice if desired. Once all of the individual slices for a given frame are received by the rendering computer, the image data for each slice are deserialized into a texture buffer and assigned to a virtual 4-sided polygon, or quad, within the graphics engine’s scene. The position and orientation vectors that correspond with each slice’s image data are then used to transform the slice’s quad to the correct virtual location in the scene. UV mapping, a standard method of mapping a texture onto a 3D surface, is then used to map the imaging data to coordinates on the quad for access by the texture shader. Once all slices are de-serialized and transformed to scene space, the full set of 3D-oriented slices is rendered to stereoscopic 3D via a multipass adjustable-cutout shader program. Finally, each stereoscopic frame’s data are transmitted wirelessly to the user’s headset for display in mixed reality.

Data transfer and unpacking are implemented separately from the user interface rendering loop via a thread-pool multithreading approach, in which each slice is assigned one “parsing” and one “processing” thread, sharing two data buffers with the UI “main” thread. Rendering is performed from a “current data” buffer, while incoming data are stored in the other “receive” buffer until the TCP transfer of all slices or volumetric partitions within one frame is complete. Once the data in the receive buffer have been validated, the buffers are swapped, changing the source of image and transform data for subsequent frames displayed in the user interface. Both the 2D texture shader and 3D ray marching volumetric shader were optimized to maintain a minimum user interface framerate of 60 or 30 fps, respectively, regardless of the data acquisition rate of the scan protocol.

Free-floating stereoscopic projections were rendered using a Microsoft HoloLens device. The HoloLens is a spatially aware mixed-reality head-mounted display unit (HMD) with transparent stereoscopic displays. In this use case, the HoloLens allowed free-floating mixed-reality representations of the imaging datasets to be anchored to a user-defined position within the environment, typically next to the scanner console.

The user’s head position and orientation were used to control the transforms of the stereoscopic virtual cameras that generated the holographic projection. This allowed the user to look at different views of the rendering without an external control using natural movements. Because the virtual position of the rendering was fixed to a position in the user’s surroundings, the user could also move toward and away from it to look at image features more closely. For volumetric datasets, the impression of moving “in and out” of the rendering was achieved via adjustment of the ray marching target’s front clipping plane.

### 2.4. System Integration

Image reconstruction and rendering were performed on two dedicated computers. The computer used for reconstruction had an 8 GB NVIDIA GeForce GTX 1080 graphics card; a 10 core, 2.2 GHz Intel Xeon E5-2630 processor; and 64 GB of 2400 MHz DDR4 RAM. For holographic remoting use cases, the rendering computer had an Intel Iris Plus Graphics 650 card; a 2 core, 3.5 GHz Intel i7-7567U processor; and 16 GB of DDR4 RAM, with a Microsoft HoloLens v1 Developer Edition used as the remoting target. For direct-to-device use cases, a Microsoft HoloLens v2 Developer Edition performed all rendering tasks discretely.

A local, closed network between the MR scanner control computer, the scanner measurement computer, and the reconstruction computer was established over Ethernet via a gigabit switch with Cat 6 cable to the reconstruction computer. A second, separate network between the reconstruction computer and the target rendering system(s) was also established. The reconstruction computer and ethernet-capable rendering targets were connected to this network via Cat 5e cables. Wireless-capable rendering or Holographic Remoting targets connected via a 5 GHz 802.11ac wireless router (TP-Link Archer C7 AC1750) All rendering network connections were provided with dedicated static routes. Communication between computers within both networks was carried out using a custom-defined TCP/IP protocol in which data are serialized in a strongly ordered string and separated by a set of break characters that define various data regions within a transmission.

Separation between the two networks was maintained via the use of dedicated routing hardware and network adapters within the reconstruction machine. The reconstruction machine, being the only device present on both networks, was configured with separate fully restricted firewalls on each network adapter. Software flow control prevents routes from being established that are directed from the rendering computer to the reconstruction computer. A local 5 GHz wireless network secured with MAC address filtering and a hidden SSID was established between the router and the headset. This networking structure was implemented in order to obfuscate the system from unwanted access and to prevent introduction of unexpected information to the scanner network.

### 2.5. System Testing and Timing

The full system, including data acquisition, image reconstruction, and rendering, was tested at the MR scanner. A user wearing a HoloLens headset operated the scanner from the control computer to set up and start the scan and then viewed the rendering that appeared next to the computer. As the HoloLens is not MRI-compatible (and cannot enter Zone 4), the system was designed to be used in Zone 3 (immediately outside the scanner room at the MRI control console). Both multi-slice planar and volumetric datasets were collected. Image quality was assessed qualitatively by viewing the real-time rendering while scanning and by viewing still frames shown in a conventional tiled format after the experiment. In addition, a program in the headset recorded the user’s perspective of the experiment as a combined rendering and video capture of the environment. These recordings were replayed after the experiment to examine image and rendering quality.

The time required by each major component of the system (acquisition, reconstruction, rendering) to process one frame of data was calculated either by the fixed sequence parameters (acquisition) or by using local timing functions (reconstruction, rendering). Two timing measures were calculated: processing time and display latency. The processing time measures the time to perform the sub-steps of the reconstruction and rendering components. The reconstruction sub-steps include removing the readout oversampling, coil compression, GRAPPA, NUFFT, coil combination, and exporting final images to the rendering system. The rendering sub-steps include parsing the image data from the TCP socket, processing the position and texture data, waiting for the next frame update, and rendering and blitting to the screen.

In this system, the acquisition, reconstruction, and rendering components may be active concurrently. For example, readout oversampling occurs as each line of raw data is received. The reconstruction and rendering of one frame are also performed while data are acquired for the next frame. Additionally, final images can only be applied to the rendering and displayed to the user at timings that align with the frame rates of the display, which may not precisely align with when the processing completes. These points made it important to include a second timing metric, the display latency. The reconstruction latency measures the time between completing data acquisition and exporting the reconstructed images for rendering, and the rendering latency measures the time between when data become available on the TCP socket to when the images are displayed on the headset. The net display latency is the sum of these two and measures the time between completing the acquisition of all data for one frame and displaying that frame to the user.

Reconstruction and rendering times were measured while acquiring 500 frames of data in an imaging phantom, as described above. Timing was calculated after a 3.5 s initialization period, during which the GRAPPA weights were loaded and the mixed-reality scene was initialized.

## 3. Results

The real-time imaging and mixed-reality visualization system was successfully tested at the MRI scanner for multi-slice planar and volumetric cardiac imaging in healthy volunteers. Using a highly undersampled radial trajectory, single-slice imaging was performed at 21 fps with an in-plane resolution of 2.34 × 2.34 mm^2^ and a slice thickness of 8 mm. When imaging with two or three slices, the frame rates were 10 and 7 fps, respectively. Volumetric data were acquired in 467 and 588 ms for eight- and twelve-partition datasets, respectively, with an in-plane resolution of 2.34 × 2.34 mm^2^ and a partition thickness of 8 mm or 5 mm.

To achieve low-latency image display, the reconstruction time must be shorter than the data acquisition time and leave time for the rendering component downstream. Table 1 shows the time required for the each of the main sub-steps in the reconstruction process and the resulting reconstruction latency. The GRAPPA, NUFFT, and remove readout oversampling sub-steps are the most time-consuming. However, removing the readout oversampling occurs as data are acquired and does not exceed the data acquisition time. The reconstruction latency was therefore shorter than the total processing time. The latency was 29, 29, 29, 181, and 230 ms for the single-slice, two-slice, and three-slice planar and eight- and twelve-partition datasets, respectively. Overhead operations listed as a single sub-step included coil combination, conversion of complex floating-point values to unsigned integers, and removal of oversampling in the partition direction for volumetric data.

The time required for the rendering processing and the display latency introduced in this component of the system are given in the bottom half of Table 1. Again, the timing is broken down by primary sub-steps. Because the data can only be updated at times that align with the frame rates of the processing software and the headset, the rendering display latency is longer than the processing time. For single-slice, two-slice, three-slice, and eight- and twelve-partition volumes, the display latencies are 12, 13, 13, 41, and 56 ms, respectively.

Finally, Table 1 shows the net display latency times of the system for each of the planar and volumetric imaging conditions. In order to achieve real-time imaging, the net display latency must be shorter than the acquisition time. This was achieved for each of the imaging conditions, with display latencies of 41, 42, 42, 222, and 286 ms for single-slice, two-slice, three-slice, eight-partition, and twelve-partition datasets, respectively. It is expected that while the net processing time increases as the dataset sizes increases, the display latencies for the different planar slice conditions remain approximately the same. This is because the display latency measures the timing after the last slice in a frame is acquired.

**Figure 3:**
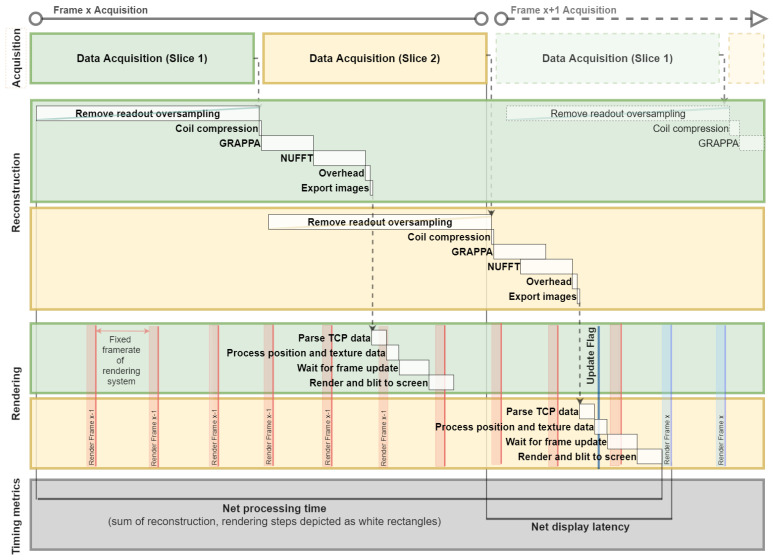
Schematic showing the relative timings of the acquisition, reconstruction, and rendering processes and their sub-steps for a two-slice planar scan. Each frame comprises two slices that are acquired sequentially. The removal of readout oversampling is performed on each line of data as it is acquired, as indicated by the diagonal bar across the sub-step rectangle. Data for the slice are buffered, followed by coil compression, GRAPPA, the NUFFT, and export of the images to the rendering system. The rendering processing includes receiving the image data over the TCP socket, parsing the data, and processing the data. Once all slices per frame are processed, as detected by an update flag, the images are rendered to the user on the next headset frame update. The net display latency is considered to be the time between when all of the data for one frame have been acquired and when that frame is displayed to the user. Note that all components of the system may be active at once, operating on different slices and/or frames of data concurrently.

Note that in Table 1, all process timings are calculated as an average per slice not frame in the case of multi-slice acquisitions. This is because the net display latency depends on processing that occurs after the acquisition of the last slice in the frame. To calculate the total processing time per multi-slice frame, the time for each sub-step should be multiplied by the number of slices. For single-slice and volumetric datasets, the processing time per slice or volume is equivalent to the processing time per frame. This timing characteristic is also illustrated in Figure 3.

**Figure 4:**
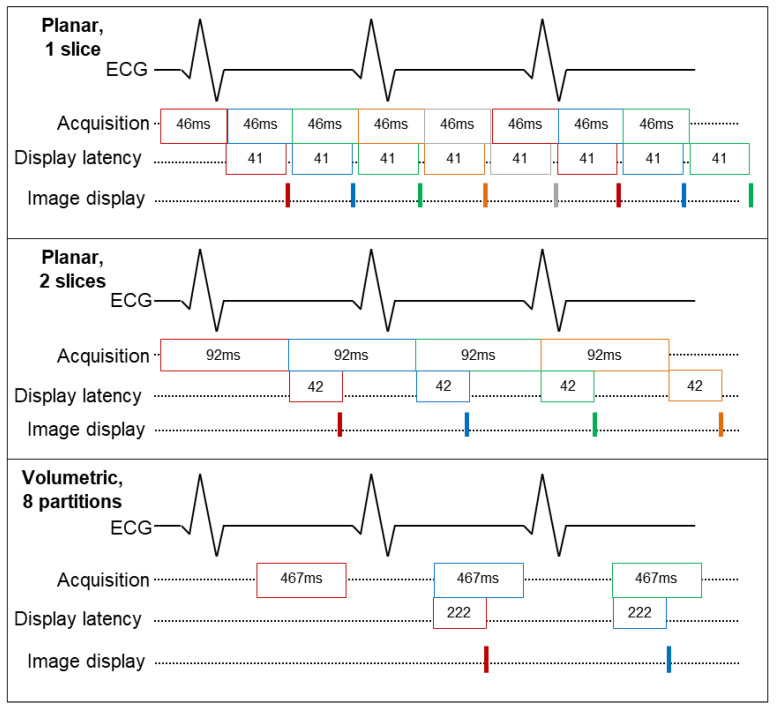
Schematic showing the time required for multiple frames of data to pass through the system for three different dataset types: (**top**) single-slice planar, (**middle**) two-slice planar, and (**bottom**) eight-partition volumetric. Planar data are acquired continuously, while volumetric data are ECG-gated. Images are displayed to the user before completing acquisition of the following frame for all cases. Different colors represent different frames.

The relative operation of the acquisition, reconstruction, and rendering components of the proposed system and their timing metrics are shown schematically in Figure 3 and Figure 4. Figure 3 shows how data for a two-slice planar acquisition move through the reconstruction and rendering sub-steps. Figure 4 shows the acquisition and net display latencies of different datasets against a schematic ECG trace to illustrate the differences in timing for gated versus un-gated acquisitions and for different numbers of planar slices. Note that for the volumetric data condition, the current version of the reconstruction pipeline only begins the reconstruction process, aside from data buffering and readout oversampling removal, once data acquisition for the next frame begins. This accounts for the display latency beginning in subsequent heartbeats. However, the images are still presented to the user before completing acquisition of the next frame of data, and it is expected that this can be corrected in future iterations of this work.

**Figure 5:**
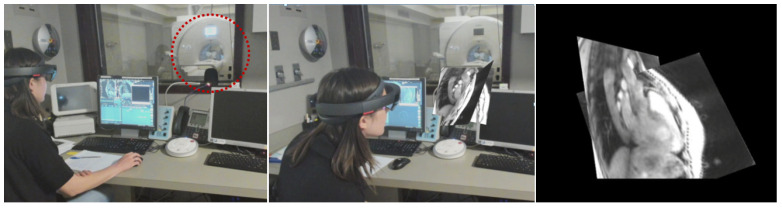
Operation of the full system at the MR scanner. (**Left**) A user wearing a mixed-reality headset sits at the scanner control computer before scanning a healthy volunteer lying in the scanner bore (red dashed circle). (**Middle**) During the scan, the user sees a multi-slice rendering of the volunteer’s heart in real-time. (**Right**) User’s view of the rendering. Note that what appears black in the image appears transparent to the user; the user sees the rendering within the natural environment. A video version of this figure is available in the Supplementary Materials.

**Figure 6:**
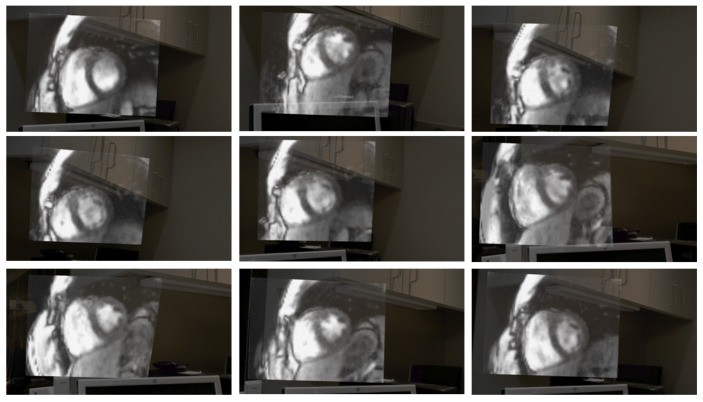
Multiple still frames from a user’s perspective of a volumetric scan through a mixed-reality headset are shown. The user moved around the rendering, changed the viewing angle, and moved toward and away from it. The stills were captured by a program in the headset that combines the rendering that is projected to the user with a video capture from the camera embedded in the headset. A video version of this figure is available in the Supplementary Materials.

Figure 5 and Figure 6 show the operation of the full system at the scanner to acquire and view multi-slice planar and volumetric datasets in the hearts of healthy volunteers. In Figure 5, two slices are oriented to capture the aortic arch and a three-chamber view. The middle panel shows a video capture of the environment with an overlay of the rendering to provide a sense of what someone looking at the scene would see. The right panel shows the user’s view of the rendering, as captured by the rendering software. Images were collected in 92 ms, with a display latency of 42 ms, enabling real-time visualization of the beating heart.

The same setup at the scanner was used acquire volumetric datasets. Figure 6 shows still frames from an eight partition acquisition from the perspective of what the user sees in the HoloLens headset, as captured by a program in the headset. The different frames show the result of the user changing positions relative to the rendering. As in the two-slice example, volumetric images were available in real-time; the acquisition took 467 ms, and the net display latency was 222 ms. Supplementary Videos corresponding to Figure 5 and Figure 6 are available online demonstrating the dynamic user movement around the visualization and giving a sense for the cardiac and respiratory motion captured by the renderings.

**Figure 7:**
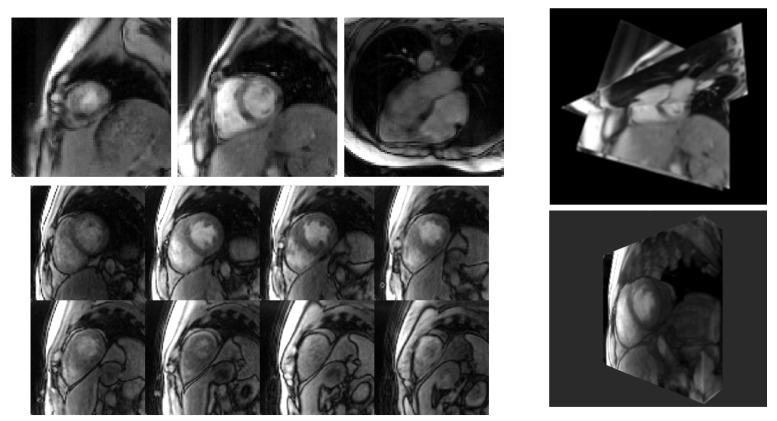
Example 2D panel display and corresponding renderings. (**Top left**) Three slices in short-axis and four-chamber views. Note that the dark bands across the ventricles in the four-chamber view are from saturation of the signal where the short-axis slices intersect. (**Top right**) Rendering of the slices in the correct spatial positions and orientations. (**Bottom left**) Panel display of eight partitions in a volumetric dataset centered over the left ventricle. (**Bottom right**) Rendering of the dataset.

Figure 7 depicts multi-slice-planar and volumetric datasets in conventional tiled formats versus corresponding renderings. When viewing static frames, the tiled format better showcases image quality in individual slices and partitions. The multi-slice planar rendering shows two short-axis slices and one four-chamber slice in the correct relative positions and orientations. The volumetric data consist of eight partitions covering the left ventricle. The rendering’s perspective and clipping plane were adjusted to emulate a view looking from the back toward to the rib cage.

## 4. Discussion

This work describes the implementation and testing of a system for real-time MRI with mixed-reality visualization. As a step toward guiding cardiovascular interventional procedures, the system is demonstrated for low-latency multi-slice and volumetric cardiac imaging in healthy volunteers. Data were acquired using a highly undersampled radial trajectory, and images were reconstructed using a GPU-parallelized implementation of through-time radial GRAPPA in the Gadgetron framework. The total display latencies were shorter than data acquisition times, enabling a user wearing a Microsoft HoloLens headset to see MR images as 3D mixed-reality renderings that are updated in real-time to reflect the current status of the person being scanned. To the best of our knowledge, this is the first system that enables real-time mixed-reality visualization of MR images at the scanner.

The future goal of guidance for interventional procedures motivated the choices of data acquisition and reconstruction methods to achieve free-breathing, ungated planar cardiac imaging at temporal resolutions exceeding rates currently deployed clinically for interventions and moving toward rates commonly reached by fluoroscopy and ultrasound. Planar imaging at 46 ms per slice, or 21 fps, was achieved using a highly undersampled radial trajectory. Multi-slice imaging was performed at 10 and 7 fps for two or three slices, respectively. The user can choose to collect images of one or more slices, trading off between temporal resolution and spatial coverage. Note that although the overall frame rate drops when additional slices are imaged, the temporal footprint of each slice remains 46 ms (the potential for motion artifacts in each slice is the same as if imaging at a single slice, but data are acquired less frequently at each position).

In this system, we additionally present rapid, beat-by-beat volumetric imaging. The long acquisition and reconstruction times of conventional techniques have limited investigations into the use of volumetric imaging in MR-guided cardiovascular interventions. Here, the undersampled radial trajectory enabled the acquisition of one volumetric dataset per heartbeat, with a temporal footprint of 467 ms or 588 ms depending on the number of partitions. However, options such as acceleration in the partition direction or use of a more efficient trajectory, such as a spiral, have previously been used in conjunction with through-time non-Cartesian GRAPPA reconstructions for faster volumetric acquisitions in the heart [23,48] and could be investigated in future work to shorten the temporal footprint further for patients with rapid heartrates.

Because the volumetric data are ECG-gated, the effective frame rate is one frame per heartbeat. One application where this type of imaging may be beneficial is during cardiac ablation, when it is important to monitor temperature changes over an area that may extend outside of one slice with rapid, online feedback. For this application, the proposed system may provide continuous planar imaging to navigate the device to the target area and then monitor temperature changes with volumetric coverage during the ablation. Modifications to the existing pipeline would need to be made for this application, including use of a real-time MR thermometry method and introduction of a respiratory motion correction module, similar to work by Ozenne et al. [25].

One of the main features of the proposed framework is its modularity. Aspects of the data acquisition and/or image reconstruction modules can be adapted for new tasks, such as ablation monitoring, without the need to modify other steps of the framework, such as the visualization. For example, other real-time cardiac imaging reconstruction approaches could be used in place of radial GRAPPA, including standard Cartesian sequences, methods available in research settings, such as nonlinear inverse reconstruction [17,26], and emerging method, such as machine learning-based reconstructions [49]. Indeed, this system could also be explored for applications where rapid, but not real-time, inline visualization capabilities may be useful. For instance, this system could be used to generate renderings of complex anatomy from which the user can plan the position of diagnostic scans. For example, in congenital heart disease patients, the MR imaging must be tailored to each patient’s unique anatomy, and a cardiac MR expert is usually needed during the exam [50]. Offline mixed-reality and virtual-reality tools are already being tested for surgical planning in this patient population [51], and an online tool could be developed for potentially more precise or faster image positioning.

The modularity of the framework also permits the display device and the user interface to be updated while maintaining the same acquisition and reconstruction. We plan to use the presented pipeline as the core framework to develop intervention-specific implementations, including adding user interface modules. Ideally, a visualization platform for interventional imaging would be capable of fusing and displaying inputs from a variety of sources within the interventional suite. While in this minimal demonstration of the pipeline architecture, the scanner is the only data source, the swap-based data transmit and receive chains allow multiple data sources to be processed independently and asynchronously. This approach ensures that user interaction with the visualization remains smooth regardless of delays elsewhere in the pipeline. Additionally, the architecture presented here is based on commodity networking hardware and protocols and is flexible enough to support further expansion as research needs change. With appropriate networking and security, a similar system could be used to transmit data to a remote physician for outside consultation or collaborative interventional procedures. The system could additionally be expanded into a multi-user system [37,47] in which all users interact with the same mixed-reality rendering.

## 5. Limitations and Future Work

The data acquisition, image reconstruction, and visualization methods presented in this work serve as an initial test and development framework, demonstrating that it is feasible to collect and present MR images of the beating heart in mixed reality at spatiotemporal resolutions required for interventional procedures. One possible use configuration may be in-room guidance, in which an operator standing next to the scanner wears an MR-compatible version of the headset, and the mixed-reality display replaces the traditional monitor display. Alternatively, this system could be used with remote-controlled robotic devices, allowing the user to stand outside of the scanner room and operate the device from a distance. However, its utility in either configuration relies on successful parallel development of MR-compatible equipment, such as needles, catheters, and robotic systems [52,53,54,55], as well as the design and validation of an MR-compatible mixed-reality headset by hardware vendors.

An imaging pipeline for interventional guidance should also allow the user to flexibly adjust the slice position during a scan, ideally directly from the rendering. However, there are two obstacles that make this feature challenging in our current implementation. First, while non-Cartesian GRAPPA can be used to achieve high-quality, real-time cardiac images, new GRAPPA weights must be calculated at new slice positions. In this work, the weights are calculated from a separate calibration scan, which would introduce a pause in the imaging each time the slice is moved. In future work, a self-calibrated accelerated scan may be used to eliminate the need for a separate calibration step [56,57,58,59]. Alternatively, one could use a method such as non-Cartesian SENSE [16]. Such a method would not require a new calibration scan but would require a longer inverse calculation based on a previously acquired coil sensitivity map.

A second obstacle is that control of the slice position from the rendering requires feedback from a user interaction with the rendering to the scanner control computer, which is typically not allowed from an external device in order to ensure regulatory compliance. In collaboration with our scanner vendor, we have recently presented preliminary work towards this end [60] showing initial feasibility.

In order to improve the usability of the current visualization, we have added interactions through hand gestures to allow the user to rotate, zoom, and adjust the window/level of the rendering [61]. However, the final design of a comprehensive user interface and set of interactions—whether through hand gestures, voice commands, gaze and head position, or a combination of these—should be conducted in collaboration with interventionalists, and this development would likely be a significant step that should be carried out in parallel with further development of the system.

## 6. Conclusions

This work demonstrates a real-time system that enables users to view a mixed-reality rendering of cardiac MR images with a display latency that is shorter than the data acquisition time. The system combines accelerated data acquisition using an undersampled radial trajectory; fast, GPU-accelerated through-time radial GRAPPA reconstruction; and rapid rendering methods. Planar images are acquired in 46, 92, and 138 ms/frame for one, two, or three slices, respectively, (21, 10, and 7 fps), and volumetric data are acquired in 467 or 588 ms/volume for eight or twelve partitions, respectively. The reconstruction and rendering steps are performed more rapidly than the acquisition, with a total display latency of 41, 42, 42, 222, and 286 ms, respectively, for each of these conditions. The final renderings are displayed using a mixed-reality headset to provide a different, potentially more intuitive way to view volumetric and multi-slice planar MR images. The system operates in real-time next to the scanner, opening up future opportunities for the use of guiding interventional procedures.

## Figures and Tables

**Table 1:** Time required by the main components in the system to process one frame of data for different dataset sizes. The net display latency is the sum of the total reconstruction latency and the total rendering display latency. In order to achieve real-time imaging, the net display latency must be shorter than the acquisition time. The reconstruction latency is the sum of the listed main sub-steps and overhead steps. It captures the processing time between when all data have been buffered and when the image is exported for display. Note that the readout oversampling is removed from each line of data as it is acquired, so this step is not included in the latency. The total rendering display latency is the sum of the sub-steps listed. Process timings are calculated as an average per slice or volume because the net display latency depends on processing that occurs after the acquisition of the last slice or volume per frame.

	Planar (Slices)			Volumetric (Partitions)	
	1	2	3	8	12
*Remove Readout Oversampling*	*8.94 (±1.28)*	*8.74 (±1.17)*	*8.74 (±1.21)*	*95.3 (±7.35)*	*112 (±7.64)*
Coil Compression	0.478 (±0.149)	0.486 (±0.109)	0.480 (±0.110)	5.08 (±0.889)	6.08 (±1.14)
GRAPPA	10.7 (±1.49)	10.7 (±1.51)	10.7 (±1.49)	119 (±3.49)	159 (±4.23)
NUFFT	15.3 (±1.76)	15.3 (±1.79)	15.3 (±1.81)	46.3 (±2.73)	54.8 (±2.52)
Export Data to TCP	0.750 (±0.461)	0.720 (±0.429)	0.727 (±0.555)	3.69 (±1.16)	3.47 (±0.908)
Overhead	1.60 (±0.203)	1.60 (±0.236)	1.60 (±0.178)	5.79 (±0.956)	6.86 (±0.881)
**Total Reconstruction Latency (ms)**	**29.1 (±2.42)**	**29.1 (±2.37)**	**29.1 (±2.42)**	**181 (±5.36)**	**230 (±5.54)**
Parse TCP Data	2.24 (±2.68)	2.28 (±2.30)	2.35 (±1.95)	32.0 (±12.5)	46.7 (±14.3)
Process Position and Texture Data	1.53 (±2.76)	1.60 (±1.04)	1.33 (±6.71)	-	-
Wait for Frame Update (vSync)	5.25 (±4.66)	5.65 (±5.90)	6.09 (±5.74)	6.23 (±5.27)	5.88 (±4.50)
Render and Blit to Screen	3.02 (±0.789)	2.90 (±0.737)	2.93 (±0.756)	3.10 (±1.05)	3.04 (±1.22)
**Total Rendering Display Latency (ms)**	**12.2 (±7.42)**	**12.5 (±8.20)**	**13.0 (±7.71)**	**41.3 (±17.2)**	**55.6 (±18.3)**
**Net Display Latency (ms)**	**41.3 (±10.2)**	**41.6 (±10.8)**	**42.1 (±10.1)**	**222 (±22.6)**	**286 (±24.0)**
**Acquisition (ms)**	46	92	138	467	588

## Data Availability

The data presented in this study are not publicly available due to restrictions in the Institutional Review Board approval.The source code for the implemented through-time radial GRAPPA reconstruction in the Gadgetron and Unity visualization system are available publicly on GitHub at https://github.com/CWRU-MRI/Realtime-MRI-Visualization. External researchers are welcome to contact the authors for assistance reproducing or expanding our implementation.

## Supplementary Materials

The following are available online at https://www.mdpi.com/article/10.3390/jimaging7120274/s1, Video S1: Video of full system operating at the MRI scanner. Video S2: Video of a user observing a volumetric dataset in real-time at the MRI scanner.
