# Supplementary material for: A System for Real-Time, Online Mixed-Reality Visualization of Cardiac Magnetic Resonance Images
Source: J Imaging. 2021 Dec 14;7(12):274. doi: 10.3390/jimaging7120274 (PMC8709155; doi:10.3390/jimaging7120274)
Supplement: Supplementary file 1 [file jimaging-07-00274-s001.zip › SupplementaryVideoCaptions.pdf]

Supplementary Video S1: Video of full system operating at the MRI scanner

A healthy volunteer is lying in the scanner bore, and the user operates the scanner from the control computer. Once the scan starts, images appear in the real-time display window on the control computer, and the rendering appears in mixed reality to the user's right. The panel on the left shows what the user sees. Note that black appears transparent to the user. Further, in this capture, the object is moving to compensate for the user's motions to look at it; this is showing that the object has to move relative to a fixed, real-world origin into order to appear stationary to a moving user. Because the user is moving in the real world relative to the global origin, the rendering also has to move. But, through the headset, the user sees a rendering apparently fixed in space. This video is captured using a third webcam that overlays the rendering onto the camera captured video of the user and the environment. This gives a better sense of what someone looking at the scene would see.

Supplementary Video S2: Video of a user observing a volumetric dataset in real-time at the MRI scanner

A user wearing a mixed-reality headset acquires volumetric data on a healthy volunteer and observes the real-time rendering. The user moves "in and out" of the rendering, or views from the base to the apex and back, and then looks at the chest wall and the spine.
